# Precision medicine phase II study evaluating the efficacy of a double immunotherapy by durvalumab and tremelimumab combined with olaparib in patients with solid cancers and carriers of homologous recombination repair genes mutation in response or stable after olaparib treatment

**DOI:** 10.1186/s12885-020-07253-x

**Published:** 2020-08-10

**Authors:** Jean-David Fumet, Emeric Limagne, Marion Thibaudin, Caroline Truntzer, Aurélie Bertaut, Emilie Rederstorff, Francois Ghiringhelli

**Affiliations:** 1 Department of Medical Oncology, Center GF Leclerc, 1 rue du Professeur Marion, 21000 Dijon, France; 2 Research Platform in Biological Oncology, Dijon, France; 3 GIMI Genetic and Immunology Medical Institute, Dijon, France; 4 University of Burgundy-Franche Comté, Dijon, France; 5 UMR INSERM 1231, Dijon, France; 6 Department of Epidemiology and Biostatistics, Georges François Leclerc Center, Dijon, France

**Keywords:** PARP inhibitors, Immune checkpoint inhibitors, Olaparib, Durvalumab, Tremelilumab, Homologous repair

## Abstract

**Background:**

Tumors with deficient homologous repair are sensitive to PARP inhibitors such as olaparib which is known to have immunogenic properties. Durvalumab (D) is a human monoclonal antibody (mAb) which inhibits binding of programmed cell death ligand 1 (PD-L1) to its receptor. Tremelimumab (T) is a mAb directed against the cytotoxic T-lymphocyte-associated protein 4 (CTLA-4). This study is designed to evaluate the efficacy of combination of olaparib, durvalumab and tremelimumab in patients with a solid tumors with a mutation in homologous gene repair.

**Methods:**

This phase II study will assess the efficacy and safety of olaparib/D/T association in patients (*n* = 213) with several types of solid cancers (breast cancer, ovarian cancer, pancreatic cancer, endometrial cancer, prostate cancer and others) with at least one mutation in homologous repair genes (*BRCA1, BRCA2, PALB2, ATM, FANCA, FANCB, FANCC, FANCE, FANCF, CHEK2, RAD51, BARD1, MRE11, RAD50, NBS1, HDAC2), LKB1/STK11, INPP4B, STAG2, ERG, CHEK1, BLM, LIG4, ATR, ATRX, CDK12*). Good performance status patients and corresponding to specific inclusion criteria of each cohort will be eligible. STEP1: Patients will receive olaparib 300 mg BID. In absence of progression after 6 weeks of olaparib, they will follow STEP 2 with olaparib and immunotherapy by durvalumab (1500 mg Q4W) + tremelimumab (75 mg IV Q4W) during 4 months and will further pursue durvalumab alone until disease progression, death, intolerable toxicity, or patient/investigator decision to stop (for a maximum duration of 24 months, and 36 months for ovarian cohort). Primary endpoint is safety and efficacy according to progression-free survival (PFS) of olaparib + immunotherapy (durvalumab + tremelimumab) during 4 months followed by durvalumab alone as maintenance in patients with solid cancers and in response or stable, after prior molecular target therapy by olaparib; secondary endpoints include overall survival (OS), disease control rate (DCR), response rate after 6 weeks of olaparib, safety of olaparib/durvalumab/tremelimumab association. Blood, plasma and tumor tissue will be collected for potential prognostic and predictive biomarkers.

**Discussion:**

This study is the first trial to test the combination of olaparib and double immunotherapy based on molecular screening.

**Trial registration:**

NCT04169841, date of registration November 20, 2019

## Background

With the development of cost effective and rapid technology of genome sequencing, precision medicine becomes a new way to think oncology. Current targets involve mainly tyrosine kinases but DNA repair machinery could also be targetable. Some of DNA repair aberrations have been associated with sensitivity to platinum and poly adenosine diphosphate [ADP]–ribose polymerase (PARP) inhibitors like olaparib, suggesting that treatment with a PARP inhibitor (PARPi) may exploit a synthetic lethal interaction, in the presence of alteration of the homologous repair pathway. PARP is involved in multiple aspects of DNA repair, and the PARP inhibitor olaparib has recently been approved for treating ovarian cancers with BRCA1/2 mutations [1, 2]. Similar results were also observed with clinical benefit of olaparib in BRCA2 mutated pancreatic cancer and in BRCA1/2 mutated breast cancer [3, 4]. In addition, a report in the New England Journal of Medicine using a high-throughput, next-generation sequencing assay in prostate cancer showed the detection of genomic alteration in genes involved in homologous repair pathway *BRCA2, ATM, BRCA1, PALB2, CHEK2, FANCA*, and *HDAC2*, is associated with response to olaparib [5]. Recently, TOPAR-B confirmed these results [6]. Thus demonstrating the clinical validation of the usage of precision medicine to position PARP inhibitors like olaparib based on molecular analysis rather than on tumor type.

Similarly, checkpoint inhibitors targeting PD-1 or PD-L1 have demonstrated an efficacy in multiple cancer types. Currently some biomarkers could be used to predict checkpoint inhibitor efficacy in a tumor type agnostic manner. High level of mutation results in high number of neoantigens and antitumor immune response, providing the rational to use immunotherapy to target such tumor types. Microsatellite instability gives rise to a high number of mutations and is associated with good response to immunotherapy whatever the cancer type. A large cohort of patients treated with pembrolizumab in multiple cancer types shows that high tumor mutation burden (TMB) is associated with response, no matter cancer type [7]. Additional DNA damage response (DDR) machinery dysfunction like deficit in homologous repair may lead to accumulation of mutations. After receiving anti-PD-1/PD-L1 treatment, patients with DDR deficiencies had a higher response rate compared to patients without these deficiencies.

Preclinical studies showed DNA damage promotes neoantigen expression [8]. PARPi-mediated catastrophic DNA damage induces accumulated chromosome rearrangements, generates neoantigens and thus increases mutation burden [9]. It is possible that increased DNA damage by PARPi would expand neoantigen expression, leading to greater immune recognition of the tumor. PARPi is also associated with immunomodulation. The PARPi talazoparib increases the number of peritoneal CD8^+^ T cells and natural killer cells and increases production of interferon (IFN)-γ and tumor necrosis factor–α (TNF)- α in a *BRCA1*-mutated ovarian cancer xenograft model [10]. Some preclinical reports also underline the capacity of PARP inhibitors to induce Type I IFN and enhance both MHC and PD-L1 expression. Hence, addition of PARPi to immune checkpoint blockade could complement the clinical benefit of immune checkpoint inhibition. Preclinical data underline a putative synergy and recent data of MEDIOLA study suggest a possible synergy between olaparib and durvalumab [11].

So, we propose to generate a clinical trial based on precision medicine to evaluate the use of immunotherapy in patients with different type of cancers with altered homologous recombination repair genes and without progression after prior targeted therapy.

The objective of this study is to determine whether the combination of olaparib plus durvalumab and tremelimumab could be effective in homologous repair deficient (HRD) tumors.

## Methods

### Study design

**Fig. 1:**
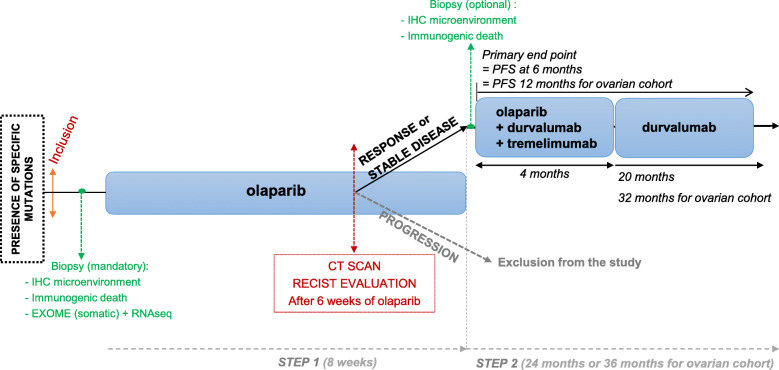
Study design

This is a multicenter, open label, non-randomized, prospective, phase II study. Patients with mutations in homologous recombination repair genes will be identified through NGS, performed at investigator sites. Analysis will be performed prior to study, as part of a clinical study or in accordance with the usual practice at the investigator site. Study design is depicted in Fig. 1.

### Study objectives

The primary objective of the study is to evaluate the efficacy of olaparib + immunotherapy (durvalumab + tremelimumab) during 4 months, followed by durvalumab alone as maintenance treatment in patients with solid cancers and in response or stable, after prior molecular target therapy by olaparib based on molecular sequencing (mutation in homologous gene repair).

Study secondary objectives are:

1. To evaluate 6-month disease control rate (DCR) for all cohorts, except ovarian cohort which will be evaluated at 12-months.
2. To evaluate 1 and 2-year PFS and overall survival (OS).
3. To evaluate response rate after 6 weeks of olaparib therapy (STEP 1).
4. To evaluate safety of molecular therapy by olaparib at the end of STEP1.
5. To evaluate safety of molecular therapy (olaparib) + immunotherapy (durvalumab + tremelimumab) and immunotherapy in maintenance (durvalumab alone).

The study exploratory objective is to evaluate the immunogenic effect of olaparib (modification of immune infiltrate and immunogenic cell death markers on biopsy performed before and after olaparib treatment).

### Study endpoints

The primary endpoint is progression free survival (PFS) 6 months after the initiation of immunotherapy for all cohorts, with the exception of ovarian cohort evaluated at 12 months. PFS is defined as the time from the date of first dose of immunotherapy to the date of progression or death from any cause. Progression will be defined using iRECIST criteria.

Secondary endpoints are:

1. Disease control rate, including stable and responsive disease, evaluated 6 months after immunotherapy initiation for all cohorts except ovarian cohort, evaluated at 12 months.
2. Overall survival (OS) evaluated at 12 and 24 months after immunotherapy initiation defined by the time of the first immunotherapy dose to the date of death from any cause.
3. Response rate evaluated by CT scan, RECIST evaluation after 6 weeks of olaparib therapy.
4. Olaparib toxicity evaluated at the end of STEP1 using CTCAE V5.
5. Olaparib + immunotherapy (tremelimumab + durvalumab) toxicity evaluated at 3, 6 and 12 months after immunotherapy initiation using CTCAE V5.

### Study population and eligibility criteria

Two hundred and seventy patients diagnosed with a solid malignancy, presenting the following histologically confirmed cancers: metastatic breast, lung, prostate, head and neck, endometrial, clear renal cell, pancreatic and urothelial cancer as well as locally advanced or metastatic ovarian cancer. Two hundred and thirteen are expected for STEP 2. Study inclusion and exclusion criteria are detailed in Tables 1 and 2.

**Table 1:** General and cohort specific study inclusion criteria

**Inclusion criteria**		
**General step 1**		
1 Patients > 18 years at time of inclusion capable of giving signed informed consent.		
2 Performance status ECOG of 0 or 1.		
3 Life expectancy ≥6 months.		
4 Body weight > 30 kg.		
5 Patients diagnosed with a solid malignancy, histologically confirmed (see cohort specific inclusion criteria below).		
6 Presence of mutation in homologous repair gene (BRCA1, BRCA2, PALB2, ATM, FANCA, FANCB, FANCC, FANCE, FANCF, CHEK2, RAD51, BARD1, MRE11, RAD50, NBS1, HDAC2), LKB1/STK11, INPP4B, STAG2, ERG, CHEK1, BLM, LIG4, ATR, ATRX, CDK12). Homozygote or heterozygote mutations and loss of heterozygosity of the second allele accepted^a^.		
7 At least one lesion measurable as defined by standard imaging criteria for the patient’s tumor type (RECIST v1.1) that can be accurately assessed at baseline and suitable for repeated assessment.		
8 Patients must have normal organ and bone marrow function.		
9 Female and male with adequate contraception method.		
10 For all oral medications patients must be able to comfortably swallow capsules.		
11 Patients affiliated to a social security regimen or beneficiary of the same according to local requirements.		
**General step 2**		
12 CT Scan evaluation after 6 weeks of olaparib should present response or stable disease as defined by RECIST v1.1 criteria.		
**Inclusion criteria**		
**Cohort specific**		
**Breast cancer**^**bd**^ - 2nd line and after	**Lung cancer**^**bd**^ - Non-small cell lung cancer. - Must have progressed after at least a first line with platinum based therapy.	**Head and neck cancer**^**bd**^ - Must have progressed after at least a 1st line with platinum based therapy.
**Metastatic endometrial cancer**^**bd**^ - Progression after one prior systemic, platinum-based chemotherapy.	**Clear cell renal cancer**^**bd**^ - Must have progressed after at least a line with anti-angiogenic agent.	**Pancreatic cancer**^**bd**^ - Must have progressed after at least a line with FOLFIRINOX regimen and/or Gemcitabine based chemotherapy.
**Ovarian cancer**^**ce**^ - Must have received at least one and no more than two lines of prior platinum-containing therapy and progressed after the most recent platinum therapy in a platinum-sensitive timeframe (more than 6 months from the last dose of platinum before randomization).	**Urothelial cancer**^**bd**^ - -2nd line and after.	**Prostate cancer**^**bd**^ - Documented evidence of metastatic castration resistant prostate cancer (mCRPC). - Ongoing therapy with LHRH analog or bilateral orchiectomy. Must have progressed on prior new hormonal agent (enzalutamine or abiraterone) and taxane chemotherapy.

^a^ With patient consent, exome sequencing of tumor and constitutive DNA should have been already performed during prior patient medical care, either as part of a clinical study or in accordance with the usual practice at investigator site, and should comprise the mandatory gene list indicated in inclusion criteria 6

^b^ Metastatic; ^c^ Locally advanced or metastatic; ^d^ ≥2nd treatment line; ^e^ 2nd or 3rd treatment line

**Table 2:** Study exclusion criteria

Exclusion criteria	
**Step 1**	
1 Patients involved in GUIDE2REPAIR study planning and/or conduct.	
2 Patients with EGFR, BRAF, ROS1 mutation or ALK rearrangement with lung small cell cancer and are not eligible.	
3 Patient eligible for another study of AstraZeneca Participation in another clinical study with an investigational product within 2 months prior to first olaparib administration.	
4 Administration of any anticancer therapy ≤21 days prior to the first dose of olaparib or 5 times its half-life, whichever smaller.	
5 Any unresolved toxicity NCI CTCAE Grade ≥ 2 from previous anticancer therapy^a^.	
6 Any concurrent chemotherapy, IP, biologic, or hormonal therapy for cancer treatment^b^.	
7 Radiotherapy treatment to more than 30% of the bone marrow or with a wide field of radiation within 4 weeks of the first dose of study drug^c^.	
8 Major surgical procedure within 28 days prior to inclusion and patients must have recovered from any effects of any major surgery IP.	
9 Patients unable to swallow orally administered medication and patients with impairment of gastrointestinal (GI) function or GI disease that may significantly alter drug absorption of oral drugs.	
10 History of allogenic organ, bone marrow or double umbilical cord blood transplantation^d^.	
11 Active or prior documented autoimmune or inflammatory disorders.	
12 Uncontrolled intercurrent illness or patient considered at medical risk due to a serious, uncontrolled medical disorder or psychiatric illness/social situation that would limit study compliance, substantially increase risk of incurring AEs or compromise patient’s ability to give written informed consent.	
13 Currently taking medications with known risk of prolonging the QT interval or inducing “torsades de pointes”.	
14 Concomitant use of known strong or moderate CYP3A inducers.	
15 Resting ECG indicating uncontrolled, potentially reversible cardiac conditions, as judged by the investigator or patients with congenital long QT syndrome.	
16 Patients with myelodysplastic syndrome/acute myeloid leukemia or with features suggestive of MDS/AML.	
17 History of another primary malignancy^e^.	
18 Patient with symptomatic central nervous system (CNS) metastases who are neurologically unstable or require increasing doses of corticosteroids or local CNS-directed therapy to control their CNS disease.	
19 History of active primary immunodeficiency and immunocompromised patients.	
20 Active infection.	
21 Current or prior use of immunosuppressive medication within 14 days before inclusion.	
22 Administration of live attenuated vaccine within 30 days prior to the first dose of IP.	
23 Female patients who are pregnant or breastfeeding or male or female patients of reproductive potential not willing to employ effective birth control.	
24 Known allergy or hypersensitivity to any of the study drugs or excipients.	
25 Prior treatment with any PARP inhibitor including olaparib or immunotherapy.	
**Step 2**	
Patients should not enter the study if any of the exclusion criteria from STEP 1 and the following criteria for STEP 2 are fulfilled: 26 Patient with progression observed on CT scan performed after 6 weeks of olaparib (STEP 1).	

^a^ Except alopecia, ototoxicity, vitiligo, and laboratory values defined in inclusion criteria. Patients with Grade ≥ 2 neuropathy will be evaluated on a case-by-case basis after consultation with study physician. Patients with irreversible toxicity not reasonably expected to be exacerbated by treatment with olaparib or durvalumab or tremelimumab may be included only after consultation with study physician

^b^ Concurrent use of hormonal therapy for non–cancer-related conditions (e.g., hormone replacement therapy) is acceptable

^c^ Non-palliative radiotherapy within 21 days prior the first dose of study drug or within 6 weeks for therapeutic doses of MIBG or craniospinal irradiation. Palliative radiotherapy (which would be < 30% of the bone marrow) to non-target lesions is allowed

^d^ Patient with allogenic stem cell transplant within 3 months prior the first study dose of Olaparib are not eligible. Patient with myeloablative therapy with autologous hematopoietic stem cell rescue within 8 weeks of the first study drug dose are not eligible. Patients receiving any agent to treat or prevent graft-versus host disease ‘GVHD) post bone marrow transplant are not eligible for this trial

^e^ Except for: malignancy treated with curative intent and with no known active disease ≥5 years before the first dose of IP and of low potential risk for recurrence; adequately treated non-melanoma skin cancer or lentigo maligna without evidence of disease; adequately treated carcinoma in situ without evidence of disease and history of leptomeningeal carcinomatosis

### Investigational products

Olaparib, durvalumab and tremelimumab will be supplied by AstraZeneca.

### Study procedures

All patients respecting all eligibility criteria, namely presenting mutations in homologous repair genes will be included. Regimens sequence is presented in Fig. 1. Study will be divided into two successive steps:

- *Step 1*
  After 6-weeks of olaparib, all patients from STEP 1 will have a CT scan.
- *Step 2*
  Depending on CT scan results, two situations will occur:
  1. Patients with stable disease or partial response will follow olaparib + immunotherapy by durvalumab + tremelimumab during 4 months and then continue with durvalumab alone until progression or until 24 dosing maximum of durvalumab for all cohorts except for ovarian cohort with a maximum of 36 dosing.
  2. Patient with progression will be withdrawn from the study and treated according to standard care.

#### Ancillary studies

Ancillary studies will be conducted to evaluate the immunogenic effect of study treatments. Before any procedure, patients should consent to participate to the exploratory studies by signing a specific informed consent form.

### Treatment doses and regimens

#### Olaparib

Patients will be administered olaparib study treatment tablets orally at a dose of 300 mg bid.

The maximum duration of treatment with olaparib is 8 weeks, corresponding to the duration of STEP 1, + 4 months, the duration of the initial part of STEP 2. The initial dose of 300 mg bid will be made up of 2 × 150 mg tablets bid. All doses of study treatment should be taken at the same time every day approximately 12 h apart.

#### Durvalumab + tremelimumab combination therapy

Patients in the durvalumab + tremelimumab combination therapy treatment will receive durvalumab (1500 mg Q4W) in combination with tremelimumab (75 mg IV Q4W) for up to 4 doses/cycles each, followed by durvalumab 1500 mg Q4W until confirmed PD (progressive disease), unacceptable toxicity, consent withdrawal, or occurrence of events leading to treatment discontinuation. With a maximum of 24 dosing of durvalumab (36 dosing for ovarian cohort).

Tremelimumab will be administered first. Durvalumab infusion will start approximately 1 h (maximum 2 h) after the end of the tremelimumab infusion. Standard infusion time for each is 1 h.

The first durvalumab monotherapy dose at 1500 mg Q4W will be 4 weeks after the final dose of durvalumab in combination with tremelimumab. (Cycle 5). After 20 cycles, durvalumab will be stopped for all cohorts, except for the ovarian cohort for a maximum of 32 months with durvalumab.

All patients will be followed for disease progression and survival until 24 months after the end of treatment.

### Treatment duration and criteria for retreatment

**Fig. 2:**
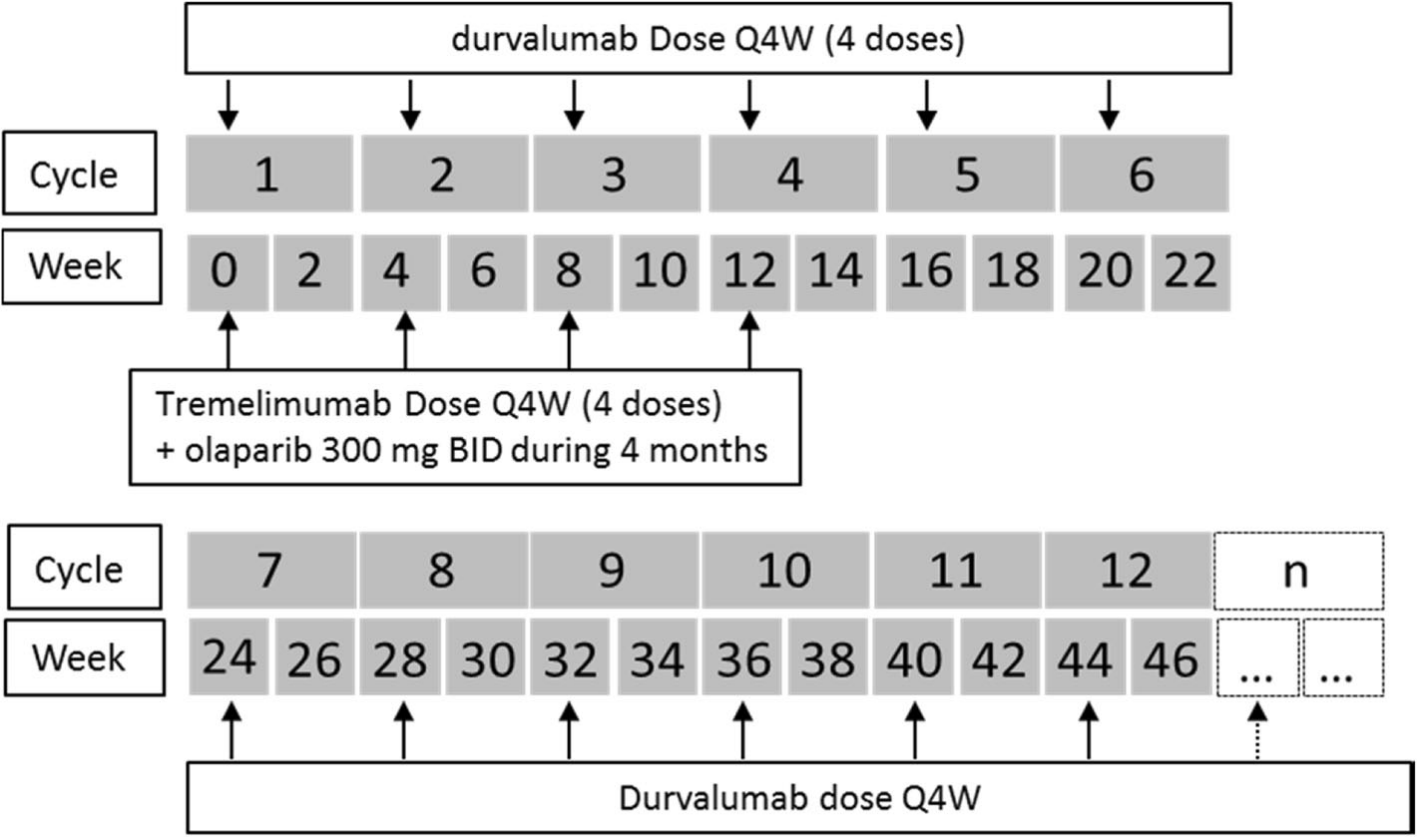
Olaparib + durvalumab + tremelimumab combination therapy dosing schedule

Treatments will be administered on Day 1 for up to 4 months for olaparib + durvalumab + tremelimumab and then from day 1 of month 5 for up to day 1 of month 24 (all cohorts) or month 36 (only for ovarian cohort) for durvalumab alone or until confirmed PD, unacceptable toxicity, consent withdrawal or occurrence of events leading to treatment discontinuation (Fig. 2). Patient will have a maximum of 24 doses of durvalumab, except for ovarian cohort that will reach a maximum of 36.

Patients with rapid tumor progression or with symptomatic progression requiring urgent medical intervention will not be eligible to continue durvalumab ± tremelimumab treatment.

For all patients who are treated through progression or patients who achieve disease control [i.e., CR, PR, or SD] at 12 months and restart treatment upon evidence of PD during follow-up, the investigator should evaluate whether patients have any significant, unacceptable or irreversible toxicities that might indicate that continuing or restarting treatment would not further benefit the patient. Patients who progressed during the first 4 months, during combination of durvalumab and tremelimumab are not allowed to be retreated.

Patients meeting the retreatment criteria below, will respect the same treatment guidelines followed during the initial 12-month treatment period, including the same dose and treatment frequency of durvalumab + tremelimumab without olaparib and the same schedule of assessments. The only exception will be the ancillary study, tumor biopsies won’t need to be collected a second time.

Patients who meet the criteria for retreatment may only receive retreatment once.

- Patients may not undergo retreatment if:
  Patients have evidence of PD during the portion of the regimen concerning the combination period of durvalumab + tremelimumab. Olaparib will not be restarted.
- Patients may undergo retreatment as described below:
  - Patients who complete the 4 dosing cycles of the combination olaparib + durvalumab and tremelimumab portion of the regimen (with clinical benefit per Investigator judgment), but subsequently have evidence of PD during the durvalumab monotherapy, with or without confirmation according to RECIST 1.1, may restart treatment with the combination, if there are 4 months between last cycle of first combination treatment and progression.
  - Patients who achieve and maintain disease control (i.e., CR, PR, or SD) through to the end of the 12-month treatment period may continue treatment with durvalumab alone until evidence of PD, with or without confirmation and according to RECIST 1.1, during follow-up.

For all patients who restart treatment with the combination as well as patients who continue Durvalumab alone, the investigator should ensure:

- Patients do not have any significant, unacceptable, or irreversible toxicities that might indicate that continuing treatment would not further benefit the patient.
- Absence of clinical symptoms or signs indicating clinically significant disease progression accompanied by a decline in WHO/ECOG performance status to > 1.
- Absence of rapid disease progression or threat to vital organs or critical anatomical sites requiring urgent alternative medical intervention.
- Patient still fulfills eligibility criteria for this study. Patients must also agree to fill an additional consent to restart durvalumab + tremelimumab combination therapy.
- If applicable, retreatment will be possible:

During the retreatment period, patients in the durvalumab + tremelimumab combination therapy group will resume durvalumab dosing at 1500 mg Q4W with 75 mg of tremelimumab Q4W for 4 doses/cycles each. Patients will then continue with durvalumab monotherapy at 1500 mg Q4W, beginning at Week 16, 4 weeks after the last dose of combination therapy, until disease progression or up to a total of 9 additional doses/cycles with the final dose at Week 48 (Month 12). Olaparib will not be restarted.

### Withdrawal and discontinuation criteria

At any time, subjects are free to withdraw from the study (investigational product and assessments), without prejudice of further treatment (withdrawal of consent). The reason(s) for withdrawal and the presence of any AEs will be investigated.

Patients who withdraw consent for further participation will not receive any further investigational product or further study observation, with the exception of follow-up for survival, which will continue until the end of the study unless the patient has expressly withdrawn his or her consent to survival follow-up.

Subject will be considered lost to follow-up only if no contact has been established by the time the study is completed such that there is insufficient information to determine the subject’s status at the time.

Reasons that will lead to permanent investigational product discontinuation are detailed in Table 3. Any patient who has not yet shown objective radiological disease progression at withdrawal from investigational product should continue to be followed as per RECIST 1.1.

**Table 3:** Criteria for permanent discontinuation of investigational product

Discontinuation criteria - Investigational product	
1 Patient weight falls to 30 kg or less.	
2 Withdrawal of consent to participate in the study or lost to follow-up.	
3 Withdrawal of consent for further treatment with investigational product.	
4 Adverse event that, in the opinion of the investigator or the sponsor, contraindicates further dosing.	
5 Patient who will probably meet one or more exclusion criteria at study entry, to whom pursuing investigational therapy might constitute a safety risk.	
6 Pregnancy or intent to become pregnant.	
7 Any AE that meets discontinuation criteria.	
8 Grade ≥ 3 infusion reaction to durvalumab or tremelimumab.	
9 Patient non-compliance that, in the opinion of the investigator or sponsor, warrants withdrawal.	
10 Initiation of alternative anticancer therapy including another investigational agent.	
11 Confirmation of PD and no benefit from treatment with olaparib or durvalumab + tremelimumab.	
12 Bone marrow findings consistent with MDS/AML.	

### Statistical analyses

Continuous variables will be summarized using descriptive statistics, i.e. number of subjects with available data (N), mean, median, standard deviation (S.D.), 25–75% quartiles (Q1-Q3) and range. Continuous variables could be transformed as categorical variable using median or using conventional cut-off from bibliography or clinical practice. If required comparison using Chi square (or exact Fisher test) or Student T test (or Wilcoxon Mann and Whitney) tests will be done. Categorical variables will be described by and percentages. The number of missing data will be described.

Each cohort will be described and analyzed separately.

#### Sample size calculation

Sample size was determined using an A’Hern’s single stage design. The primary endpoint is progression PFS. It will be evaluated at 6 months (except for ovarian cancer with an evaluation at 18 months given the results of olaparib in SOLO-2 trial). The hypotheses are the following:

- one-sided alpha risk =10%, a power = 90%,
- the expected rate of patients stable or in response after 6 weeks of olaparib is 80%,
- the expected rate of patients non-evaluable the primary endpoint is 5%,
- P0 and P1 are determined using hypotheses detailed above.

P0 is the highest level of inefficacy for which the new treatment will be rejected (maximal inefficacity). P1 defines the minimum required level of efficacy. The design of the trial focuses on demonstrating that this level is plausible given that the trial results and the efficacy is greater than the first proportion, P0. Taking into account these hypotheses: 270 patients will be included in the study, and at least 213 are required for statistical analyses. At the end the study, analysis of the primary endpoint will be performed on evaluable patients who were in response or stable after 8 weeks of olaparib.

#### Analysis of safety endpoint(s)

Safety analyses will be performed on the safety-evaluable population, defined as all subjects treated with at least one dose of investigational product.

In each cohort, toxicities and grades will be described according to the type of event at each cycle.

The global following data will also be given:

- The number and percentage of patients with at least one adverse event over the study period,
- The number and percentage of patients with at least one grade 3 or 4 adverse event over the study period,
- The number and percentage of patients with at least one serious adverse event over the study period,
- The number and percentage of patients with at least one adverse event leading to treatment premature stop over the study period.

In case a patient experience more than one toxicity, the toxicity with the highest grade will be considered.

Time until grade 3–4 toxicity will be determined using the Kaplan Meier method. Patients without toxicities will be censored.

#### Analysis of efficacy endpoint(s)

Efficacy analyses will be performed in modified intent-to-treat population (ITT) population ie, all patients with a 6-month evaluation (12-month for ovarian cohort) and at least one dose of durvalumab + tremelimumab. Analyses will be repeated in the per protocol population (patients who had received all the planned doses).

6-month PFS rate (12 month-rate in the ovarian cohort) will be determined using the Kaplan Meier method. 95% Confidence interval (CI) will be provided. Patients alive without progression will be censored.

Median follow-up will be estimated using the reverse Kaplan Meier method.

PFS and Overall survival will be estimated using the Kaplan Meier method. Six-month, 1-year, 2-year rates as well as median OS and PFS will be reported with their 95% CI.

Disease control rates at 6 months will be determined and described with its 95% binomial CI. The same method will be applied to describe the response rate after 6 months of olaparib in each cohort.

All statistical analysis will be performed with SAS 9.4. In case of comparison, tests will be two-sided.

#### Exploratory analyses

Analysis of immunogenicity will be performed based on IHC analysis. We will analyze the micro environment with T Cells and PD-L1 expression. Moreover, we will analyze immunogenic death with HMGB1 and LC3. These parameters will be compared before and after a target therapy to evaluate its impact on microenvironment.

#### Interim analyses

An Independent Data Monitoring Committee (IDMC) will be established for Guid2Repair trial. The IDMC will consist of designated sponsor, 2 or 3 experts in oncology and 1 statistician. The IDMC will be responsible for an independent evaluation of the safety for the patients participating in the clinical trial. Additional IDMC meetings could be required if more than 15% of ongoing patients have experienced an adverse event ≥ grade 3.

No interim analyses are planned for efficacy.

### Ethical and regulatory requirements

The study will be performed in accordance with ethical principles that have their origin in the Declaration of Helsinki, including Decree no. 2016–1537 of 16/11/ 2016 on research involving human subjects, and are consistent with ICH/Good Clinical Practice, and applicable regulatory requirements Patient Data Protection; in France CNIL.

Before carrying out research on humans, the sponsor is required to submit the project to the opinion of one of the competent institutional ethical committee (Comité de protection des Personnes CPP) and to the regulatory authority (ANSM).

Prior to the implementation of the research on a person, eligible subject will be fully informed by the investigator during the consultation and after a period of reflection sufficient written informed consent form will be collected.

The information will also include information on data handling in accordance with the revised French Data Protection regulations including European General Data Protection Regulation N° 2016/679.

## Discussion

Among all solid tumors, a subgroup of patients presents a deficiency in homologous recombination repair genes. This abnormal DNA repair system sensitizes these tumors to PARP inhibitors. There is a strong rational to combine PARP inhibitors and immune checkpoint inhibitors. Indeed, PARP inhibitors induce DNA damage causing neoantigen increase, leading to a reinforced recognition by the immune system. Moreover, PARP inhibitors increase PD-L1 expression. In 2017, Lee et al have shown that anti-PD-L1 and olaparib combination is safe [12]. Recently, phase 2 MEDIOLA has shown promising results in BRCA mutated breast cancer with a control rate of 80% at 12 weeks [13]. In the light of these results, immunogenic target therapy with olaparib in combination with immune checkpoint inhibitors could act synergistically and might be a promising treatment in tumors with deficiencies in homologous recombination. Furthermore, blood, plasma and tumor tissue will be collected and assessed for potential prognostic and predictive biomarkers. Study is ongoing and the first patient was included in February 11th 2020.

## Data Availability

Not applicable.

## Abbreviations

Aes: Adverse events
CI: Confidence interval
CTLA-4: Cytotoxic T-Lymphocyte-Associated protein 4
DCR: Disease control rate
DDR: DNA damage response
HRD: Homologous repair deficient
IDMC: Independent Data Monitoring Committee
ITT: Intent-to-treat population
NGS: Next Generation Sequencing
OS: Overall survival
PARP: Poly-ADP-Ribose Polymerase
PD-1: Programmed Death – 1
PD-L1: Programmed Death Ligand 1
PFS: Progression-Free Survival
Q4W: Every 4 weeks
TMB: Tumor mutation burden
